# Hydrogen Transport, Viscoelastic Drift, and Multiscale Characterization Framework for Elastomeric Seals Under High-Pressure Hydrogen

**DOI:** 10.3390/polym18101198

**Published:** 2026-05-14

**Authors:** Nitesh Subedi, Md Monjur Hossain Bhuiyan, Alfredo Becerril Corral, Omkar Gautam, Md Ariful Islam, Zahed Siddique

**Affiliations:** School of Aerospace and Mechanical Engineering, University of Oklahoma, Norman, OK 73019, USA

**Keywords:** hydrogen exposure, elastomer degradation, hydrogen transport, dynamic mechanical analysis, nuclear magnetic resonance, µXCT, high-pressure exposure

## Abstract

High-pressure hydrogen exposure may induce transport and diffusion–relaxation–controlled changes in elastomeric sealing materials that differ from conventional fluid aging. Hydrogen uptake through solution–diffusion processes can lead to swelling, redistribution of molecular mobility, viscoelastic evolution, and, under certain conditions, cavitation or microvoid formation during decompression, which may affect long-term sealing performance. This review compiles experimental results for commonly used elastomers, including Nitrile Butadiene Rubber (NBR), hydrogenated nitrile butadiene rubber (HNBR), Fluoroelastomer (FKM), Ethylene Propylene Diene Monomer (EPDM), and silicone, and summarizes reported ranges of hydrogen diffusivity, solubility, and permeability under high-pressure conditions. These transport characteristics are compared with mechanical and microstructural observations obtained from Dynamic Mechanical Analysis (DMA), Nuclear Magnetic Resonance (NMR), decompression testing, and micro-computed tomography (µXCT) imaging. Available evidence suggests that hydrogen-induced changes are predominantly governed by physical processes, including swelling, plasticization-like mobility changes, and constraint redistribution, while extensive chemical degradation of the polymer backbone is generally limited under clean hydrogen conditions. Materials with similar conventional mechanical properties may, therefore, exhibit different hydrogen uptake, viscoelastic response, and resistance to decompression damage. Conventional single-point mechanical tests, such as tensile measurements, may not fully capture the time-dependent viscoelastic evolution relevant to sealing performance. This work proposes a multiscale characterization framework integrating transport, viscoelastic, molecular, and microstructural analysis for more reliable evaluation of elastomers in hydrogen service, supporting improved qualification strategies for high-pressure hydrogen systems.

## 1. Introduction

Elastomeric sealing materials are widely used in high-pressure gas containment systems because they maintain conformal contact, accommodate deformation, and provide leak-tight interfaces under complex loading conditions. In high-pressure hydrogen environments, however, elastomers experience transport-driven and time-dependent changes that differ from conventional fluid exposure. Hydrogen penetrates polymer networks through solution–diffusion processes and may accumulate in free-volume regions or interfacial domains, influencing swelling and mechanical response [1]. These effects can influence mechanical stability and sealing-force retention even when extensive chemical bond scission is not observed under typical clean hydrogen exposure conditions.

In contrast to metallic components, whose hydrogen compatibility is often evaluated using fracture- or fatigue-based criteria, elastomer degradation is governed primarily by coupled transport, swelling, and relaxation phenomena. Repeated hydrogen uptake and release during pressure cycling may promote volumetric expansion, stress redistribution, and gradual microstructural damage. Rapid gas decompression (RGD) can further induce internal cavitation or cracking when pressure histories exceed the diffusion–relaxation capability of the material [2]. As a result, elastomeric seals are often among the most performance-sensitive components in high-pressure hydrogen systems. The broader transition toward hydrogen-based energy technologies is driven by thermodynamic, environmental, and infrastructure-scale decarbonization requirements, as discussed in recent reviews of hydrogen production and deployment pathways [3,4].

Hydrogen-induced changes in elastomers depend on transport parameters, network architecture, filler reinforcement, and time-dependent mechanical behavior [1]. These factors influence swelling magnitude, modulus evolution, compression-set development, and internal damage morphology during and after hydrogen exposure. Conventional mechanical characterization methods, such as tensile strength, hardness, and compression set, are widely used to evaluate elastomer performance for engineering practice; however, these approaches are inherently limited for hydrogen environments, as they may not fully explain the underlying mechanisms responsible for changes in these properties. These tests are typically performed under quasi-static or equilibrium conditions and do not capture the time-dependent coupling between hydrogen transport, swelling, and viscoelastic relaxation that governs material response under high-pressure exposure. Similar conventional properties do not necessarily reflect hydrogen transport or time-dependent behavior. This limitation highlights the need for characterization approaches that can resolve both transport behavior and time-dependent mechanical response.

In recent studies, techniques such as DMA, NMR, and microstructural imaging have been increasingly used to probe elastomer behavior across multiple length and time scales. These methods provide complementary information on viscoelastic response, molecular mobility, free-volume structure, and internal damage evolution. Integration of these techniques with transport measurements offers a multiscale framework for understanding hydrogen–elastomer interactions and for establishing more reliable criteria for material selection and qualification in high-pressure hydrogen systems.

Despite significant progress in understanding hydrogen transport and mechanical response in elastomers, existing studies largely report these phenomena independently without establishing a unified mechanistic linkage. Unlike prior review studies that primarily report hydrogen transport, mechanical behavior, and degradation phenomena separately, the present work provides a unified multiscale interpretation framework linking hydrogen diffusion, viscoelastic relaxation, and damage evolution in elastomeric sealing materials. In particular, this study integrates transport parameters such as diffusivity and solubility with polymer network dynamics and filler-induced heterogeneity to explain pressure-dependent behavior under high-pressure hydrogen environments. Furthermore, the role of filler–polymer interactions, dual sorption mechanisms, and diffusion–relaxation mismatch is critically analyzed to provide a physically consistent interpretation of hydrogen-induced behavior across different elastomer systems. This integrated perspective enables a more predictive understanding of hydrogen-induced damage mechanisms, which is not sufficiently addressed in the existing literature.

This review compiles published experimental results for commonly used sealing elastomers, including nitrile butadiene rubber (NBR), hydrogenated nitrile rubber (HNBR), fluoroelastomers (FKM), ethylene–propylene–diene monomer (EPDM), and silicone-based systems. Emphasis is placed on hydrogen transport measurements, viscoelastic property evolution, and microstructural observations obtained using permeation and desorption methods, DMA, NMR, and micro-X-ray computed tomography (µXCT). The objective is to organize available data in a consistent, measurement-based framework that supports comparative evaluation of elastomer behavior under high-pressure hydrogen exposure.

## 2. Hydrogen Service Environments Relevant to Elastomers

High-pressure hydrogen service conditions define the environmental range within which elastomeric seals are evaluated. Sealing materials may be exposed to pressures from several megapascals to above 90 MPa, together with variable temperatures and repeated pressurization–depressurization cycles [5,6]. These conditions influence hydrogen uptake, swelling, viscoelastic response, and mechanical stability during service [6,7]. In practical systems such as hydrogen storage vessels, refueling infrastructure, and valve sealing applications, these conditions directly govern sealing reliability and long-term leakage performance. Under elevated pressure, hydrogen dissolves into polymer networks through solution–diffusion processes, producing volumetric expansion and redistribution during depressurization [8,9,10]. During pressure changes, internal concentration gradients develop over characteristic diffusion time scales driven by chemical potential differences [11]. However, elastomer response depends on pressure history and decompression rate, as discussed in Section 3.3 [10,12,13]. Hydrogen distribution may also become heterogeneous, with localized accumulation that can contribute to cavitation during decompression [14].

Temperature modifies both transport kinetics and mechanical response. Increasing temperature accelerates diffusion and stress relaxation, while lower temperatures increase stiffness and may promote hydrogen retention near the glass-transition region (Tg) [9,15]. Reduced mobility at low temperature can increase susceptibility to decompression-induced damage [14,16]. Accordingly, hydrogen exposure conditions should be reported explicitly. Hydrogen service conditions are defined by pressure, temperature, dwell time, decompression rate, and cycling history, which provide the necessary context for interpreting transport, mechanical, and microstructural behavior discussed in later sections [5,10,12].

### 2.1. Key Environmental Parameters

Evaluation of elastomers under hydrogen exposure requires systematic control of environmental variables. Pressure governs hydrogen uptake and swelling, temperature controls diffusion and relaxation, dwell time determines saturation, and decompression rate strongly influences rapid gas decompression behavior [8,10,12]. Cyclic pressurization introduces repeated swelling and desorption that may contribute to progressive mechanical evolution [7,17]. Post-exposure conditioning is equally important since hydrogen desorption and viscoelastic recovery continue after decompression. Measured properties may therefore depend on the time between pressure release and testing [9,13]. Table 1 summarizes representative environmental parameters and their relevance to hydrogen–polymer interaction and degradation behavior.

### 2.2. Elastomer Materials and Baseline Property Considerations

Elastomers used in hydrogen sealing applications are evaluated based on transport behavior, mechanical stability, and viscoelastic response under defined exposure conditions [5,12]. Transport parameters (diffusivity, solubility, permeability) are determined through permeation or desorption methods, while mechanical behavior is assessed using tensile testing, compression set, hardness, and DMA [10,15]. These properties should not be interpreted independently, as swelling and mechanical response depend on network structure, free volume, and filler–polymer interactions. Materials with lower hydrogen uptake may still exhibit greater swelling depending on network compliance [9,19,24]. Accordingly, hydrogen response depends strongly on formulation variables such as crosslink density, filler content, and plasticizer level rather than polymer type alone [12,24]. Compatibility should therefore be evaluated at the formulation level under controlled conditions rather than inferred solely from polymer classification [5,12,22].

## 3. Hydrogen–Elastomer Interaction Mechanisms

Elastomer behavior in hydrogen environments can be interpreted in terms of mechanisms governed by solution–diffusion processes and influenced by pressure, temperature, decompression history, and formulation [10,25]. Table 2 provides a concise overview of key degradation mechanisms and their corresponding effects on seal performance under hydrogen exposure. Each mechanism should be interpreted in the context of transport parameters (D, S) and mechanical resistance to cavitation.

### 3.1. Sorption, Diffusion, Desorption

Gas transport in elastomeric materials is commonly interpreted using the solution–diffusion framework, in which permeability $\left(P\right)$ is expressed as the product of diffusivity $\left(D\right)$ and solubility $\left(S\right)$, i.e., P=S⋅D. Within this framework, gas uptake and transport are governed by two coupled processes: dissolution of hydrogen into the polymer matrix and subsequent diffusion through the free-volume structure of the elastomer [26]. At the molecular level, solubility is controlled by polymer–gas interactions and local free-volume distribution, whereas diffusivity depends on chain mobility, crosslink density, and network constraints.

However, elastomer systems—particularly filled and highly crosslinked materials—frequently deviate from ideal Fickian behavior. These deviations arise from heterogeneous free-volume distributions, filler–polymer interfacial regions, and constrained chain dynamics, which can introduce multiple transport pathways and time-dependent diffusion behavior. In such systems, dual-mode sorption (combining Henry-type dissolution in the polymer matrix and Langmuir-type trapping at filler interfaces), reduced or pressure-independent diffusivity, and sub-diffusive transport have been widely reported. These effects are often linked to fractional free volume and its evolution during swelling, which directly influences diffusivity through changes in chain mobility and segmental relaxation. A more quantitative interpretation of diffusion in elastomers can be obtained using free-volume-based transport models, such as the Vrentas–Duda free-volume framework, which relates diffusivity to the fractional free volume and segmental mobility of the polymer network. In this approach, diffusivity is expressed as an exponential function of available free volume and temperature, reflecting the coupling between molecular transport and polymer chain dynamics [27]. As hydrogen sorption increases free volume and modifies segmental relaxation behavior, diffusivity may evolve dynamically rather than remaining constant. This provides a mechanistic basis for observed deviations from Fickian transport and highlights the importance of coupling between sorption-induced swelling and diffusion kinetics in elastomer systems [27].

As a result, permeability in elastomers cannot always be described using a single constant diffusivity, and transport behavior must be interpreted in the context of free-volume-controlled diffusion, interfacial constraints, and diffusion–relaxation coupling, particularly under high-pressure hydrogen exposure conditions. Under equilibrium conditions, the concentration of dissolved hydrogen in the elastomer is often approximated by Henry-type behavior. In practice, however, hydrogen sorption in elastomers—particularly in filled systems—often deviates from ideal Henry-type behavior and exhibits dual-mode characteristics. This behavior arises from two distinct contributions: (i) dissolution of hydrogen within the polymer matrix governed by Henry’s law and (ii) localized adsorption or trapping at filler–polymer interfaces, free-volume clusters, or microstructural heterogeneities.

Experimental observations in filled elastomers, including carbon-black-reinforced NBR systems, indicate that filler particles introduce additional sorption sites and interfacial regions that can retain hydrogen, leading to increased apparent solubility and reduced effective diffusivity due to trapping–release processes [21,24,26]. As a result, hydrogen transport cannot be described solely by bulk diffusion, but instead reflects a coupled mechanism involving matrix diffusion and interfacial trapping [26,28].

This dual-mode transport behavior contributes to non-Fickian sorption kinetics, delayed desorption, and spatially heterogeneous hydrogen distribution within the elastomer, all of which are critical in determining swelling behavior and susceptibility to decompression-induced damage. Accordingly, for idealized conditions in the absence of significant trapping or interfacial effects, hydrogen sorption can be approximated using classical thermodynamic relations, and the equilibrium hydrogen concentration can be expressed as:

C_0_ = S · p

where $C_{0}$ is the equilibrium hydrogen concentration in the polymer, S is the solubility coefficient, and p is the applied hydrogen pressure. This relation describes equilibrium hydrogen uptake, whereas time-dependent transport behavior is governed by diffusion processes. The diffusion coefficient characterizes molecular transport through the free-volume structure of the elastomer [8,10]. These parameters depend strongly on polymer chemistry, crosslink density, filler content, temperature, and morphology. To describe the time-dependent evolution of hydrogen transport beyond equilibrium solubility considerations, a transient diffusion framework is required. The transient evolution of hydrogen concentration within elastomeric materials is governed by Fick’s second law, which relates temporal changes in concentration to spatial gradients arising from diffusion processes.

$$\frac{\partial C}{\partial t}=D\nabla^{2}C$$

where C is the local hydrogen concentration as a function of position and time.

For geometries relevant to sealing applications, such as cylindrical O-rings and thick sections, analytical solutions of Fickian diffusion are commonly employed in desorption and permeation studies to extract effective diffusivity and total hydrogen uptake from sorption and desorption measurements. These solutions further indicate that during rapid decompression, steep concentration gradients develop near the material surface during decompression, resulting in high initial desorption flux and non-equilibrium internal pressure distributions, which are directly linked to decompression-induced damage mechanisms.

Despite this formulation, deviations from ideal Fickian behavior are frequently observed for many laboratory exposure conditions, but deviations are frequently reported in filled or highly crosslinked elastomers. Multi-stage sorption, delayed desorption, or sub-diffusive behavior can occur due to heterogeneous morphology, filler–matrix interfaces, constrained chain regions, or multiple free-volume environments [25,28]. Molecular-scale studies also indicate that hydrogen may exist in different mobility states, leading to transport heterogeneity even when macroscopic uptake appears approximately Fickian.

Additionally, hydrogen sorption influences transport behavior through changes in free volume and diffusion pathways, contributing to transient dimensional variation [18]. Because transport and mechanical behavior are coupled, interpretation of hydrogen-exposed elastomers requires combined analysis of transport measurements and mechanical characterization [10,17]. Transport parameters should be considered together with exposure conditions, including pressure, temperature, specimen geometry, and formulation. In particular, the relationship between characteristic diffusion time and pressurization–decompression time governs whether the response remains near equilibrium or develops internal concentration gradients that may influence swelling, relaxation behavior, and decompression response [8,25]. Quantitative comparison of diffusivity, solubility, and permeability across elastomer systems is therefore necessary to interpret differences in hydrogen uptake, desorption kinetics, and susceptibility to decompression-related damage. Representative transport data for selected elastomers are summarized in Table 3. Under high-pressure hydrogen exposure, the accumulation of dissolved gas within the elastomer provides a direct link between transport behavior and failure mechanisms such as RGD. During pressure release, retained hydrogen may generate internal gas pressure within free-volume regions or microvoids. When this internal pressure exceeds the mechanical resistance of the polymer network, cavitation and damage can occur.

The severity of RGD is therefore governed by the interplay between solubility and diffusivity: higher solubility promotes greater gas uptake, while lower diffusivity limits the rate of gas release during decompression. This combination increases the likelihood of transient internal pressure buildup, linking transport parameters directly to decompression-induced failure behavior discussed in the following section.

In clean hydrogen environments, swelling is primarily physical, arising from hydrogen occupying free-volume regions without significant backbone degradation [8,10]. Although hydrogen-induced changes in elastomers are predominantly governed by physical processes such as swelling and mobility redistribution under clean hydrogen conditions, localized chemical effects cannot be entirely excluded. In practical hydrogen systems, trace impurities such as moisture, oxygen, or other reactive contaminants may promote localized oxidative or interfacial degradation, which should be considered when interpreting long-term service behavior. Under realistic service conditions, the presence of impurities, oxidative species, or cyclic loading may lead to limited chemical modifications, including crosslink rearrangement or interfacial degradation. Therefore, both physical and localized chemical contributions should be considered when interpreting long-term material behavior.

These mechanisms are most directly manifested through hydrogen-induced swelling and associated changes in mechanical properties. Dissolved hydrogen increases chain mobility and free volume, producing dimensional expansion and reduced network stiffness. Reported swelling is generally modest but measurable, with hydrogen uptake typically ~400–600 wt ppm for NBR and EPDM and lower values for FKM and HNBR, depending on formulation [9,19,26]. Swelling increases with pressure, temperature, and exposure time, but does not scale directly with hydrogen content, reflecting the influence of crosslink density, filler reinforcement, and network compliance [8,12].

Measured dimensional changes include contributions from mechanical and thermal effects, and corrected values reflect hydrogen uptake governed by transport parameters and network structure [8,10,13]. Post-decompression recovery is time-dependent due to continued desorption and viscoelastic relaxation, requiring clear reporting of measurement timing. Hydrogen sorption also modifies viscoelastic response, with DMA typically showing reduced storage modulus and increased damping consistent with mobility redistribution [15,22,24].

In filled systems, weakening of filler–matrix interactions may further increase stress relaxation, while unsynchronized diffusion and relaxation can promote internal void formation. These effects are formulation dependent. Higher crosslink density and filler reinforcement improve dimensional stability, whereas plasticized or weakly reinforced systems show greater swelling and reduced resistance to decompression damage [12,13,26].

**Figure 1:**
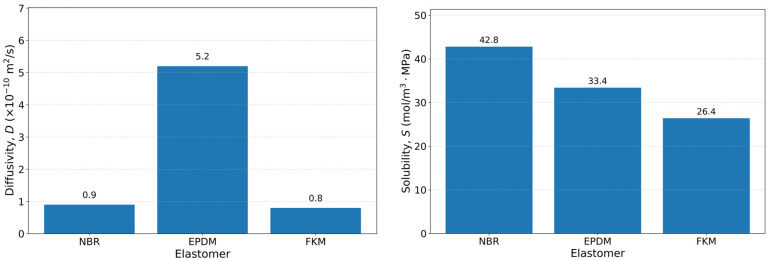
Hydrogen diffusivity (D) and solubility (S) of selected elastomers under comparable high-pressure hydrogen exposure conditions (data adapted from Jeon et al., Polymers, 2022 [29]).

These data are further illustrated in Figure 1, highlighting comparative trends among elastomer families. The observed variation in diffusivity values for a given elastomer (e.g., EPDM) may reflect differences in formulation, crosslink density, and experimental conditions and should, therefore, be interpreted as representative ranges rather than intrinsic material constants.

However, this variability can be more rigorously interpreted by considering the underlying structural and interfacial factors governing hydrogen transport. Hydrogen transport behavior in elastomers is strongly influenced by polymer network structure, filler content, and interfacial interactions. Increased crosslink density reduces free volume and restricts molecular mobility, leading to lower diffusivity, whereas loosely crosslinked or plasticized systems exhibit higher diffusivity due to enhanced chain mobility.

The presence of fillers introduces additional complexity by modifying both diffusion pathways and sorption behavior. At the molecular scale, filler–polymer interactions create immobilized chain segments and interfacial layers that act as physical crosslinks, reducing chain mobility and altering effective diffusivity. In addition, fillers increase tortuosity and introduce trapping sites at interfacial regions, which can retain hydrogen and lead to dual-mode transport behavior. As a result, hydrogen transport in filled elastomers reflects a coupled mechanism involving matrix diffusion and interfacial trapping rather than purely bulk diffusion.

Furthermore, filler characteristics such as particle size, surface area, and dispersion state influence the extent of polymer chain immobilization and modify effective transport pathways. At elevated pressures, however, the influence of filler-induced tortuosity may diminish as bulk diffusion becomes dominant.

In addition to material-related factors, discrepancies in reported diffusivity values also arise from differences in experimental techniques, including permeation, volumetric sorption, and thermal desorption methods, which vary in sensitivity to transient versus steady-state transport behavior. Therefore, reported transport parameters should be interpreted as system-dependent rather than intrinsic material constants.

While Table 3 provides quantitative ranges, the figure highlights comparative trends and differences among elastomer families and formulations. Published high-pressure permeation and sorption studies show measurable variation in hydrogen transport parameters, which depend on pressure, temperature, specimen geometry, and compound composition. These parameters should therefore be interpreted within their specific experimental conditions rather than inferred from polymer classification alone [28,29].

Published permeation and sorption studies show measurable variation in hydrogen transport parameters among elastomer families and formulations. Diffusivity, solubility, and permeability depend on polymer type as well as pressure, temperature, specimen geometry, and compound composition, and should therefore be interpreted within the specific experimental conditions in which they are measured rather than inferred from polymer classification alone. For meaningful comparison across elastomer systems, transport parameters should be interpreted relative to underlying structural variables such as fractional free volume, glass-transition temperature (Tg), and crosslink density. Normalization of diffusivity and solubility with respect to these parameters enables more consistent comparison across materials with different formulations. For example, elastomers with similar Tg distance or free-volume fraction may exhibit comparable diffusion behavior despite differences in polymer chemistry, highlighting the importance of structure-based rather than composition-based comparison.

Hydrogen transport is also strongly temperature dependent, and the diffusion coefficient commonly follows Arrhenius-type behavior, reflecting thermally activated molecular motion within the polymer network.

$$D(T)=D_{0}\;exp\left(\frac{-E_{a}}{RT}\right)$$

where D is the diffusion coefficient, $D_{0}$ is a pre-exponential factor, $E_{a}$ is the activation energy for diffusion, R is the gas constant, and T is absolute temperature [26,28].

Increasing temperature accelerates diffusion and desorption kinetics while reducing storage modulus and increasing stress relaxation due to enhanced chain mobility [15,30]. These concurrent changes influence swelling, internal stress development, and cavity growth during decompression [12,13]. At lower temperatures, diffusivity decreases and internal gas retention may persist during pressure reduction [10]. Reduced chain mobility near the glass-transition region can promote hydrogen localization and increase susceptibility to internal stress buildup during decompression [9,15]. Hydrogen uptake may therefore produce reversible plasticization-like effects associated with increased free volume and modified relaxation dynamics [10,20]. The overall response reflects the coupled interaction of temperature-dependent transport kinetics and viscoelastic behavior rather than pressure or temperature considered independently [15,22].

Reported variations in hydrogen diffusivity and solubility across studies arise from multiple factors, including differences in specimen geometry, measurement technique (permeation vs. desorption), pressure conditions, and data interpretation methods. For example, permeation measurements typically reflect steady-state transport, whereas desorption-based methods capture transient behavior, leading to differences in extracted diffusivity values. Additionally, formulation variables such as filler loading, crosslink density, and plasticizer content introduce further variability. Therefore, discrepancies in reported values should be interpreted in the context of experimental methodology and material structure rather than treated as intrinsic material constants.

### 3.2. Rapid Gas Decompression (RGD)

Rapid gas decompression (RGD), sometimes referred to as explosive decompression, is a well-recognized failure mode in elastomeric seals exposed to high-pressure hydrogen environments [12,13]. During pressurization, hydrogen dissolves into the elastomer and redistributes through the polymer network over a characteristic diffusion time governed by transport properties and specimen geometry [8,10]. When external pressure is reduced abruptly, retained hydrogen cannot diffuse out instantaneously, producing a transient pressure imbalance within the material [12,17,31]. This non-equilibrium condition may generate localized tensile stresses, leading to nucleation and growth of gas-filled cavities when internal pressure exceeds the resistance of the elastomer network [31]. A simplified condition for cavity growth can be expressed as:

$$\Delta p=P_{int}-P_{ext\;}>\sigma_{c}$$

where $P_{int}$ represents the internal gas pressure associated with retained hydrogen, $P_{ext}$ is the external pressure after decompression, and $\sigma_{c}$ is the effective cavitation stress required for cavity expansion within the elastomer. While the simplified condition provides a first-order estimate, cavitation in elastomers is more accurately described using energy-based criteria such as the Gent model, which considers the elastic energy required for cavity growth. In this framework, cavity expansion occurs when the stored elastic energy in the deformed network exceeds a critical threshold determined by material stiffness, defect size, and network constraints. Unlike simple stress-based criteria, this approach accounts for the non-linear elasticity of elastomers and the influence of pre-existing microvoids, filler agglomerates, and interfacial debonding, which can act as initiation sites for cavity formation under decompression conditions.

From an engineering perspective, this energy-based threshold is often represented in terms of an effective cavitation resistance, $\sigma_{c}$, which provides a practical measure of a material’s resistance to cavity growth. The value of $\sigma_{c}$ depends on tensile strength, elastic modulus, crosslink density, defect population, and filler reinforcement within the compound [8,12,13,32]. Typical tensile strength of elastomers ranges from approximately 5 to 20 MPa, depending on formulation, which provides a useful estimate of cavitation resistance governing bubble growth during decompression. When internal gas pressure exceeds this threshold, cavity nucleation and growth can occur, particularly in materials with lower crosslink density or weakened filler–matrix interactions. Because elastomers in the rubbery state often exhibit relatively low resistance to tensile cavitation compared with the pressure differentials that may develop during rapid venting, even moderate transient pressure imbalances can initiate internal damage in susceptible formulations.

The severity of RGD is governed by the relationship between the decompression time ($\tau_{vent}$) and the hydrogen diffusion time within the elastomer ($\tau_{diff}$) [8,10]. The diffusion time may be approximated as

$$\tau_{diff}\sim L^{2}/D$$

where L is a characteristic diffusion length, such as seal thickness, and D is the hydrogen diffusion coefficient. When $\tau_{vent}$ ≪ $\tau_{diff}$, hydrogen cannot escape rapidly enough to relieve internal pressure gradients, producing large transient Δp values and increasing the likelihood of cavitation. When $\tau_{vent}$ is comparable to or greater than $\tau_{diff}$, pressure equilibration occurs more gradually and the risk of damage is reduced [13].

**Figure 2:**
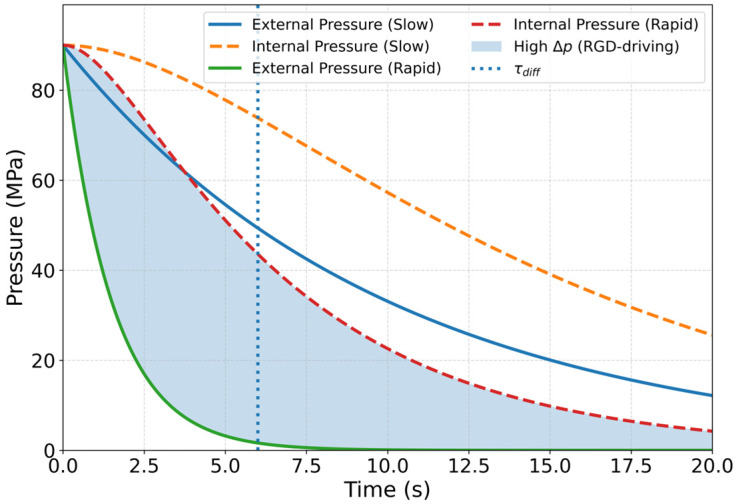
Conceptual evolution of internal and external pressures during slow and rapid decompression. The internal pressure curves are conceptual representations based on diffusion-limited behavior and are not direct experimental measurements; however, they are consistent with trends reported in high-pressure desorption and decompression studies.

Experimental observations show that RGD damage is more likely under hydrogen pressures of ~30–100 MPa, particularly when decompression occurs over short time scales. Under such conditions, diffusion times in millimeter-scale specimens (typically tens to hundreds of seconds) exceed decompression times, producing transient pressure gradients sufficient to initiate cavitation [8,13,17]. Observed damage includes internal voids, blistering, crack formation, and loss of mechanical integrity after decompression [7,12,17]. Damage severity increases with exposure pressure, dwell time, decompression rate, temperature, and cyclic loading history [31]. Therefore, resistance to RGD is governed by the combined influence of hydrogen solubility, diffusivity, and the mechanical properties of the elastomer network. While higher diffusivity may facilitate faster pressure equilibration during decompression, materials with high hydrogen uptake or lower cavitation resistance may still be susceptible to internal damage. The conceptual evolution of internal and external pressures during slow and rapid decompression is illustrated in Figure 2. The internal pressure curves represent diffusion-limited responses rather than direct measurements.

Although the pressure evolution shown in Figure 2 is conceptual, it is grounded in established diffusion-controlled decompression behavior reported in high-pressure hydrogen studies, including desorption and permeation experiments. Similar transient pressure differentials arising from diffusion–relaxation mismatch have been experimentally observed and are widely recognized as the primary driving mechanism for rapid gas decompression (RGD) damage in elastomers [8,13,17]. The figure is therefore intended to provide a physically consistent interpretation framework rather than a direct quantitative prediction.

### 3.3. Pressure Cycling and Damage Accumulation

Repeated hydrogen pressure cycling can produce progressive degradation in elastomeric seals, even in the absence of rapid gas decompression [7,12,17]. During cyclic pressurization, hydrogen sorption and desorption lead to repeated swelling and shrinkage of the polymer network, generating transient stresses and promoting viscoelastic relaxation [8,10]. Over multiple cycles, these effects may result in compression-set growth, microvoid formation, interfacial damage, and crack initiation, indicating that long-term degradation arises from cumulative transport–mechanical interactions rather than a single decompression event. Experimental studies report pressure ranges of ~10–70 MPa and cycle counts from tens to several thousand cycles, with damage severity increasing with both maximum pressure and number of cycles [12,17]. Even when individual cycles do not produce visible failure, repeated swelling–deswelling may gradually reduce modulus, increase compression set, and promote internal defect formation. In some cases, damage accumulated during cycling exceeds that produced by a single decompression at comparable pressure [7,12,17].

Microstructural damage formed during early cycles may grow or coalesce under continued loading, leading to loss of sealing force and increased leakage risk. In practical sealing geometries such as O-rings, cyclic deformation within the gland may further contribute to extrusion, surface wear, or localized damage [12]. Damage accumulation is strongly formulation dependent. Lower crosslink density or higher plasticizer content generally increases swelling and reduces resistance to cavitation, whereas filler reinforcement may improve dimensional stability but can still develop localized interfacial damage under repeated cycling. Therefore, resistance to cyclic hydrogen exposure should be evaluated at the formulation level under representative pressure histories. Evaluation of elastomer performance should consider both decompression resistance and long-term stability under repeated pressurization–depressurization conditions [12,22].

### 3.4. Viscoelastic Response and Diffusion–Relaxation Effects in Hydrogen-Exposed Elastomers

Elastomer behavior under high-pressure hydrogen exposure is governed by time-dependent mechanical response and gas uptake processes. Because elastomers exhibit time-dependent mechanical response, sealing stability depends not only on hydrogen uptake but also on the relative rates of diffusion, swelling, and stress relaxation [8,10]. Hydrogen sorption alters viscoelastic response, producing changes in stiffness, damping, and relaxation behavior without necessarily causing irreversible chemical degradation under clean hydrogen conditions [20,25,30]. Consequently, mechanical response is more appropriately interpreted in terms of transport–relaxation behavior rather than simple strength loss [33].

The coupling between transport and viscoelastic response can be further quantified using dimensionless analysis, such as the Deborah number (De), defined as the ratio of the characteristic relaxation time of the material to the characteristic diffusion time of hydrogen. When De ≪ 1, viscoelastic relaxation occurs rapidly relative to diffusion, allowing stress relaxation during gas uptake and release. In contrast, when De ≫ 1, diffusion is too slow to equilibrate pressure during decompression, resulting in stress accumulation and increased susceptibility to cavitation and rapid gas decompression damage. This framework provides a quantitative basis for interpreting diffusion–relaxation mismatch in hydrogen-exposed elastomers.

When diffusion occurs over time scales comparable to viscoelastic relaxation, stresses generated during sorption and swelling can partially relax, resulting in reversible viscoelastic softening. However, when decompression occurs under non-equilibrium conditions, internal pressure gradients may develop, as discussed in Section 3.3 [12,13]. Swelling measurements further indicate that equilibrium volume change is typically limited, whereas transient expansion during rapid decompression may be significantly larger due to retained hydrogen.

The extent of this transport–relaxation interaction is strongly formulation dependent. Elastomers with higher diffusivity (e.g., EPDM or silicone) allow faster pressure equilibration, whereas more densely packed or highly filled systems (e.g., FKM or HNBR) may retain hydrogen longer, increasing the potential for transient stress accumulation [8,10]. Localized hydrogen retention in free-volume regions or interfacial domains may further contribute to non-uniform stress development during decompression [11]. Temperature further modifies this balance by simultaneously accelerating diffusion and relaxation while reducing stiffness, thereby influencing whether the material response remains reversible or progresses toward damage [9,15].

DMA is commonly used to evaluate viscoelastic changes after hydrogen exposure through measurements of storage modulus (E′), loss modulus (E″), and damping factor (tan δ). Reported trends include reductions in rubbery modulus, increased damping, and gradual evolution of viscoelastic response during pressure cycling [15,24]. These changes are generally associated with mobility redistribution, swelling, or modification of filler–polymer interactions rather than direct chain scission and, therefore, require correlation with transport and microstructural data for interpretation [8,10,12].

Filler reinforcement further influences viscoelastic behavior. Carbon black or silica fillers form a secondary load-bearing network that contributes to stiffness at small strain. Hydrogen sorption and associated swelling may weaken filler–matrix interactions, producing increased damping and gradual stiffness reduction during cyclic loading [34]. As a result, functional degradation may occur through viscoelastic instability and loss of sealing force even in the absence of visible cracking, consistent with diffusion-controlled damage mechanisms described in Section 3.3 [7,12,13,24]. These observations indicate that optimal elastomer performance in hydrogen service requires a balance between transport and viscoelastic properties, where diffusion rates are sufficient to prevent gas trapping while network stiffness and relaxation behavior maintain sealing force under cyclic loading.

Table 4 summarizes commonly used approaches for interpreting viscoelastic and mechanical changes in hydrogen-exposed elastomers. These methods provide a framework for distinguishing reversible plasticization, diffusion-controlled stress development, and irreversible microstructural damage when evaluating DMA and related measurements.

## 4. Experimental Characterization Framework

### 4.1. Dynamic Mechanical Analysis (DMA)

**Figure 3:**
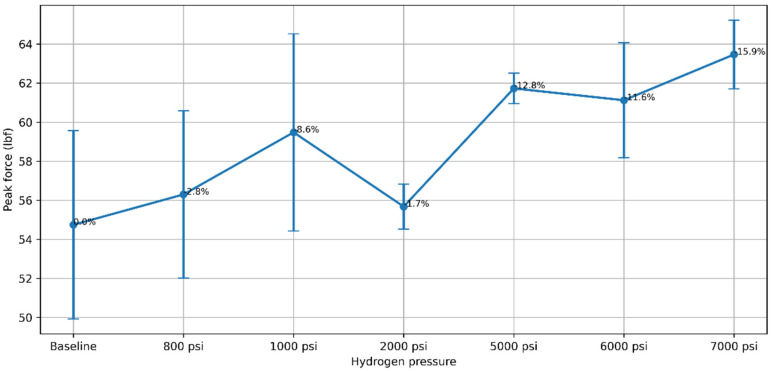
Non-monotonic peak force response of NBR seals as a function of hydrogen pressure for a fixed exposure duration (192 h) at room temperature. Error bars represent mean ± standard deviation (n ≥ 3). The observed trends illustrate the combined influence of hydrogen transport, viscoelastic relaxation, and internal stress evolution, and are presented as representative behavior consistent with the reported literature rather than a comprehensive dataset.

Mechanical and viscoelastic characterization provides direct information on the response of elastomers exposed to high-pressure hydrogen. Conventional mechanical tests, such as tensile strength, hardness, compression set, and stress relaxation, remain widely used; however, these measurements are typically performed under quasi-static or equilibrium conditions and may not capture the time-dependent interaction between hydrogen transport and mechanical response, and conventional properties may not fully reflect hydrogen-induced behavior. Conventional strength-based metrics such as peak force or tensile strength may not directly reflect sealing performance under hydrogen exposure. As illustrated in Figure 3, the peak force response of a commercial-grade 70 Shore A NBR elastomer exposed to hydrogen at different pressures for a fixed duration (192 h) at room temperature does not exhibit a monotonic trend with increasing pressure, despite identical specimen geometry and testing protocol. This deviation from the expected monotonic softening behavior indicates that hydrogen-induced mechanical response is governed by competing transport and relaxation mechanisms rather than hydrogen concentration alone. From a purely transport-driven perspective, higher hydrogen uptake at elevated pressure would be expected to promote increased swelling and softening, leading to a progressive reduction in peak force. However, the observed non-monotonic behavior indicates that the mechanical response cannot be interpreted solely in terms of hydrogen concentration. Instead, the results suggest that competing mechanisms—such as pressure-dependent diffusion kinetics, viscoelastic relaxation, and internal stress evolution during desorption—govern the effective load-bearing response. In particular, time-dependent viscoelastic recovery and relaxation processes may partially offset softening at certain conditions, while unsynchronized diffusion and relaxation can lead to transient stiffening or variability in measured force. These observations highlight that conventional strength-based metrics, including peak force or tensile response, may not directly reflect sealing performance or long-term mechanical stability under hydrogen exposure. Accordingly, characterization approaches capable of capturing time-dependent behavior, such as DMA, are necessary to resolve relaxation dynamics and more accurately assess material performance in hydrogen service environments. These trends are consistent with the coupled interaction between transport kinetics and viscoelastic response, where characteristic diffusion and relaxation time scales may differ under varying pressure conditions. These observations are intended to illustrate general trends and are not presented as a comprehensive dataset.

Figure 3 presents representative experimental observations from the authors’ ongoing experimental program to illustrate non-monotonic mechanical response under hydrogen exposure. While the dataset is not presented as a comprehensive study, the observed trends are consistent with reported behavior in hydrogen-exposed elastomers, where competing effects of swelling, viscoelastic relaxation, and internal stress evolution lead to non-linear mechanical response [8,10,24]. The figure is therefore included to support the conceptual discussion rather than to establish a generalized quantitative relationship. Accordingly, the figure should be interpreted as a physically supported illustrative representation integrated with published experimental observations rather than a standalone quantitative dataset.

DMA provides a more sensitive evaluation by measuring storage modulus (E′), loss modulus (E″), and damping factor (tan δ) as functions of temperature, frequency, and strain amplitude [12,15]. In hydrogen-exposed elastomers, these parameters reflect changes in stiffness, mobility, and filler–polymer interactions associated with hydrogen sorption and pressure cycling [33].

Because elastomers are sensitive to testing conditions, reliable interpretation requires control of deformation mode, strain amplitude, temperature, preload, specimen geometry, and post-exposure handling [15]. This is particularly important after hydrogen exposure, where swelling and continued desorption may alter the measured response. Accordingly, DMA should be interpreted together with transport behavior, decompression history, and formulation variables. Key experimental and reporting considerations are summarized in Table 5.

Hydrogen-exposed elastomers commonly show reductions in rubbery modulus and increases in damping, reflecting mobility changes and redistribution of network constraints rather than extensive chemical degradation [15,18,23]. In filled systems, hydrogen sorption may weaken filler–matrix interactions, producing stiffness drift and increased damping during cyclic loading [38,39]. These effects emphasize the importance of validating the linear viscoelastic region when comparing aged and unaged materials.

While conventional mechanical tests remain relevant for engineering qualification, they do not uniquely reflect hydrogen-induced changes. Similar post-exposure properties may correspond to different transport behavior, viscoelastic evolution, or internal damage states. DMA is therefore most effective when interpreted alongside transport and microstructural measurements [7,20,40].

**Figure 4:**
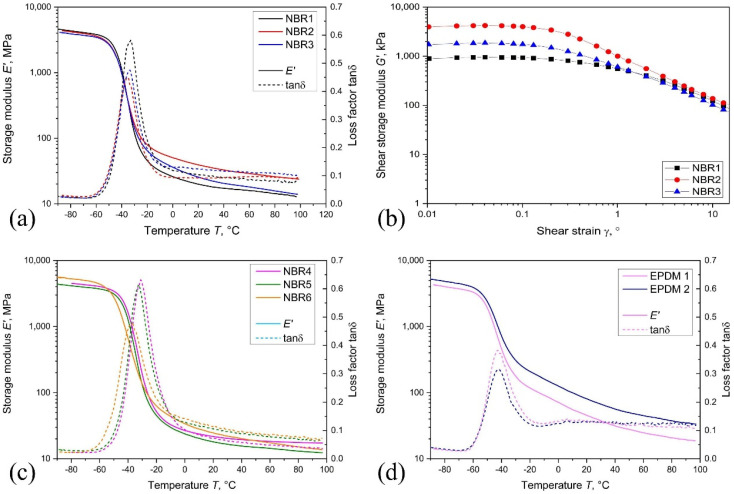
Representative DMA response of elastomer compounds developed for high-pressure hydrogen service. (**a**) Storage modulus (E′) and loss factor (tanδ) as functions of temperature for silica-filled NBR compounds. (**b**) Shear storage modulus (G′) as a function of shear strain illustrating the Payne effect in representative NBR compounds. (**c**) Storage modulus (E′) and loss factor (tanδ) as functions of temperature for carbon-black-filled NBR compounds. (**d**) Storage modulus (E′) and loss factor (tanδ) as functions of temperature for EPDM compounds. The variations in viscoelastic behavior reflect differences in filler type, filler loading, curing system, and formulation.

Figure 4 provides a representative illustration of DMA response across the glass-transition region and rubbery plateau. The storage modulus (E′) defines stiffness evolution, while tan δ reflects the temperature range of molecular mobility and energy dissipation. Variations in rubbery-plateau modulus are primarily associated with network structure and filler reinforcement, whereas shifts in tan δ indicate differences in molecular mobility and constraint distribution. These features are directly relevant to hydrogen service because they influence swelling response, stress relaxation, and sealing-force retention [15,23].

The figure highlights that elastomer performance cannot be inferred from a single modulus value. Materials with similar conventional properties may differ in damping, transition behavior, and rubbery stiffness, which can affect their response to hydrogen uptake and decompression. However, the figure represents comparative formulation behavior rather than direct hydrogen-induced degradation and should be interpreted accordingly.

In addition to formulation-dependent differences, the rubbery plateau region is particularly relevant for hydrogen service because it governs sealing-force retention under operating conditions. Variations in rubbery modulus reflect differences in crosslink density and filler reinforcement, which influence the ability of the elastomer network to resist swelling-induced softening during hydrogen uptake. Under high-pressure hydrogen exposure, materials with lower rubbery stiffness or weaker filler–matrix interactions may exhibit greater modulus reduction and accelerated stress relaxation, leading to loss of sealing force. Furthermore, shifts in transition behavior and damping characteristics suggest that different formulations may respond differently to changes in temperature, pressure, and decompression rate, even when conventional properties appear similar. These observations highlight the importance of evaluating formulation-dependent viscoelastic response when assessing elastomer performance in hydrogen environments. This formulation sensitivity is consistent with experimental observations under hydrogen exposure, where differences in filler content, crosslink density, and network structure lead to non-uniform viscoelastic evolution and damage resistance across elastomer systems [7,12,23].

To improve relevance for hydrogen qualification, DMA measurements should be performed after controlled hydrogen exposure with systematic variation in pressure, decompression rate, and cycling history. Stepwise pressure conditions and controlled decompression protocols are particularly useful for identifying transitions between reversible viscoelastic changes and more persistent stiffness drift or recovery delay. Such approaches support the development of a framework linking hydrogen transport, viscoelastic stability, and long-term sealing performance.

### 4.2. Nuclear Magnetic Resonance: Physical Basis and Measurement Principles

NMR provides molecular-scale information that complements the macroscopic viscoelastic response obtained from DMA. While DMA captures stiffness and damping at the network level, NMR probes molecular mobility, free-volume structure, and constraint distribution within the polymer, enabling detection of changes that may not be reflected in bulk mechanical measurements [15,41].

In elastomers, NMR response is sensitive to mobility, network constraints, and heterogeneity arising from crosslinks, filler interfaces, and constrained chain segments. Hydrogen sorption may modify these features through swelling and mobility redistribution, leading to measurable variations in NMR response that reflect changes in local structure and constraint environment [42,43,44]. Because elastomer networks are inherently heterogeneous, hydrogen may exhibit localized retention within free-volume regions or interfacial domains, which can influence local stress development during decompression [36,41].

Experimental studies of hydrogen-exposed elastomers generally indicate that hydrogen is predominantly physically absorbed within the polymer network. NMR observations are typically consistent with reversible mobility changes and constraint redistribution associated with swelling and plasticization-like behavior, while limited chemical modification is generally observed under clean hydrogen conditions [45,46]. Limited localized structural modification may occur under severe exposure, but these effects are generally formulation dependent.

Measurement conditions such as temperature, exposure history, and time after decompression can significantly influence NMR results. Hydrogen desorption and viscoelastic recovery may continue after exposure, and therefore, sample history should be clearly defined when comparing data across studies [36,41,47].

NMR and DMA provide complementary perspectives: DMA quantifies macroscopic viscoelastic response, whereas NMR provides molecular-level insight into mobility and constraint distribution. Correlation of these measurements, together with transport and microstructural data, supports a more consistent interpretation of hydrogen-induced changes in elastomer networks [15,41,44].

### 4.3. NMR in Hydrogen-Exposed Elastomers

Proton (^1^H) NMR is widely used to evaluate hydrogen uptake and molecular-scale behavior in elastomers. Measurements indicate that hydrogen may be distributed across multiple environments within the polymer network, including dissolved hydrogen in the matrix, confinement within free-volume regions, and localization near filler–matrix interfaces. The relative distribution of these states may evolve during sorption, diffusion, and desorption, reflecting the heterogeneous structure of elastomer networks [41,47,48].

**Figure 5:**
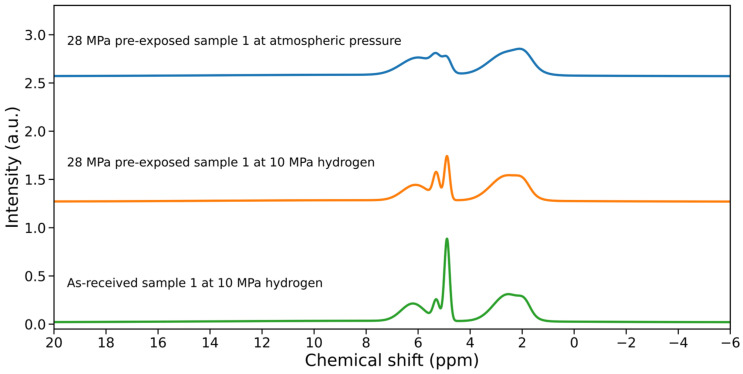
Representative solid-state ^1^H NMR spectra of an elastomer after high-pressure hydrogen exposure. Signals in the 4.5–5.5 ppm region are commonly associated with molecular hydrogen within the polymer network. Differences between pre-exposed, in situ, and recovered states reflect variations in hydrogen concentration, confinement, and desorption behavior [29,45,46,47].

Figure 5 illustrates how NMR distinguishes hydrogen populations under different exposure conditions. Variations in signal intensity reflect differences in hydrogen content and confinement within the polymer network, while comparisons between pre-exposed and post-decompression states indicate time-dependent uptake and release behavior.

The absence of strong new chemical peaks in many studies supports the interpretation that hydrogen–polymer interaction is primarily governed by physical sorption and confinement rather than extensive chemical modification. At the same time, changes in signal distribution suggest redistribution of hydrogen within free-volume regions or interfacial domains, which may contribute to localized internal pressure during decompression.

Variations in NMR response are generally consistent with changes in molecular mobility and constraint distribution, and often align with viscoelastic trends observed in DMA, such as modulus reduction and increased damping [36,43,44,48]. When interpreted together with swelling, transport, and microstructural measurements, NMR provides useful insight into mobility redistribution and structural heterogeneity, helping distinguish reversible plasticization from more persistent changes in elastomer networks [8,15,41,45].

### 4.4. Linking DMA, NMR, and Mechanical Drift in Hydrogen-Exposed Elastomers

Interpretation of hydrogen-induced mechanical changes requires correlation between macroscopic viscoelastic response and molecular-scale characterization. NMR observations indicate that hydrogen is accommodated within free-volume regions and interfacial domains, where localized retention may contribute to internal pressure gradients during decompression [12,13,45,46]. DMA captures the macroscopic consequences of these effects, including reductions in storage modulus and increases in damping associated with mobility redistribution, swelling, and modification of network constraints [15,22,24]. In filled elastomers, weakening of filler–matrix interactions may further contribute to gradual stiffness drift during cyclic loading.

Correlation of DMA and NMR results helps distinguish reversible mobility changes from more persistent structural effects. Changes dominated by increased molecular mobility are generally consistent with reversible plasticization, whereas combined changes in viscoelastic response and molecular constraints may indicate network modification or damage under repeated exposure. Because multiple mechanisms may produce similar changes in modulus or damping, interpretation based on a single technique may be uncertain [41,44,45].

This multiscale approach is particularly relevant for hydrogen sealing applications, where performance depends on maintaining contact stress over time rather than resisting immediate fracture. Linking molecular mobility with macroscopic viscoelastic behavior, therefore, provides a useful basis for evaluating hydrogen compatibility and comparing materials under different exposure conditions [8,10,12]. These relationships are summarized in Table 6, which links each characterization technique to its measured response and corresponding interpretation in hydrogen-exposed elastomers.

### 4.5. Complementary Chemical and Morphological Characterization

While DMA and NMR provide insight into viscoelastic response and molecular mobility, additional characterization is often required to distinguish reversible sorption from more persistent structural modification. Because hydrogen exposure may simultaneously influence transport, swelling, relaxation, and microstructure, complementary analytical methods are needed for consistent interpretation [12,15,41].

Fourier transform infrared spectroscopy (FTIR) is used to monitor changes in functional groups associated with oxidation, crosslink modification, or additive redistribution. In many elastomers, only minor spectral changes are observed after hydrogen exposure, suggesting that the polymer backbone remains largely intact under clean hydrogen conditions, although localized chemical changes may occur under severe conditions [20,45].

Thermal analysis methods such as DSC and TGA provide information on glass-transition behavior and thermal stability. TGA responses are often similar before and after exposure, indicating limited bulk chemical change, while shifts in Tg or relaxation behavior are typically associated with changes in molecular mobility or plasticization. Because these properties also depend on formulation variables, thermal data should be interpreted together with mechanical and transport measurements [10,15].

Swelling and sorption measurements provide direct information on hydrogen uptake and dimensional response. Interpretation requires consideration of mechanical compression, thermal effects, and continued desorption after decompression, as measured values may not represent equilibrium conditions [8,10,17].

Controlled decompression testing evaluates cavitation and internal damage under defined pressure-release conditions. Damage severity depends on decompression rate, temperature, specimen geometry, and formulation, and may be influenced by localized hydrogen accumulation within the network [12,17,32].

Microscopy techniques such as SEM and µCT enable direct observation of internal defects and damage evolution. µCT, in particular, provides three-dimensional visualization of cavities and crack networks that can be correlated with transport and viscoelastic measurements. Imaging studies indicate that weakly reinforced or plasticized materials tend to develop more extensive damage, whereas reinforced systems may limit crack propagation but still exhibit localized defects [7,20,49].

Together, these methods provide a multiscale framework linking molecular mobility, transport behavior, and macroscopic mechanical response. Combined analysis is therefore important for distinguishing reversible physical effects from more persistent structural changes and for enabling consistent comparison across materials and exposure conditions [12,15,41].

### 4.6. Hydrogen Transport and Swelling: Experimental Measurement Methods

Transport parameters, including diffusivity (D), solubility (S), and permeability (P), together with swelling behavior, provide key inputs for evaluating diffusion-related effects and assessing conditions that may lead to internal pressure gradients during decompression. Hydrogen transport in elastomers is commonly characterized using permeation measurements, sorption–desorption experiments, thermal desorption analysis, and swelling measurements under controlled conditions [8,10,17].

Steady-state permeation testing determines permeability by measuring hydrogen flux through an elastomer membrane under a defined pressure gradient. Estimation of diffusivity and solubility typically requires time-dependent uptake or release measurements. For meaningful comparison, transport parameters should be reported together with temperature, pressure, specimen geometry, exposure duration, and conditioning history, as these variables strongly influence measured behavior [8,10].

Hydrogen transport in elastomers may deviate from ideal Fickian behavior due to polymer heterogeneity, filler–matrix interfaces, and coupling with viscoelastic relaxation. Molecular simulation studies further support these observations, showing that hydrogen diffusion pathways and transport behavior depend strongly on polymer free-volume structure and chain dynamics under varying pressure conditions [50]. Consequently, transport parameters obtained from simplified or short-time measurements should be interpreted with caution, particularly for thick specimens or extended exposure durations [10,51].

Thermal desorption analysis (TDA) is widely used to quantify absorbed hydrogen following high-pressure exposure [8,17]. Accurate interpretation requires careful control and reporting of exposure conditions, specimen geometry, and decompression protocol, since hydrogen desorption may continue during handling and influence measured values [10,17,51].

High-pressure permeation systems provide complementary measurements under controlled pressure conditions. Although inert gases such as helium are sometimes used as substitutes, they do not fully reproduce hydrogen-specific sorption and swelling behavior, and direct hydrogen measurements are generally preferred [52,53].

Swelling measurements provide additional information on gas uptake through dimensional or mass change. Interpretation requires consideration of mechanical compression, thermal effects, and time-dependent desorption and relaxation, as measured values may not represent equilibrium conditions [10,20].

Transport and swelling behavior are strongly formulation dependent. Increased crosslink density and filler loading generally reduce diffusivity and swelling, whereas plasticized or weakly crosslinked systems may exhibit higher hydrogen uptake and greater susceptibility to decompression-related damage. Interfacial regions near filler particles may further influence gas localization and transport behavior [49,54].

Because transport, swelling, and relaxation occur over different time scales, consistent reporting of experimental conditions is essential. Combined interpretation of transport data with viscoelastic, molecular, and microstructural characterization provides a more reliable basis for evaluating elastomer performance in high-pressure hydrogen environments [8,12,15,41].

## 5. Importance of Polymer Characterization in Hydrogen Systems

To operationalize the proposed multiscale characterization framework, a structured evaluation methodology is required. First, hydrogen transport parameters (diffusivity and solubility) should be quantified to determine uptake and characteristic diffusion time. Second, the viscoelastic response should be evaluated using DMA to identify relaxation behavior and stiffness evolution. Third, molecular-level characterization using NMR should be used to assess hydrogen distribution and mobility states. Fourth, microstructural analysis using µXCT or microscopy should be performed to detect internal defects and cavitation. Finally, these results should be integrated to evaluate diffusion–relaxation coupling and susceptibility to decompression-induced damage, enabling formulation-level comparison and material selection.

Elastomeric seals exposed to high-pressure hydrogen may undergo time-dependent changes associated with gas uptake, swelling, viscoelastic evolution, and decompression-induced damage [8,12,20,32]. As discussed in Section 3 and Section 4, these responses are governed primarily by relaxation processes rather than fracture-controlled mechanisms typical of metallic materials. Because the magnitude and rate of these effects depend strongly on formulation, exposure history, and transport kinetics, quantitative characterization is required to evaluate mechanical stability and internal damage under controlled conditions [15,22].

Elastomer compatibility in hydrogen service cannot be assessed using a single mechanical property. A multiscale characterization approach is therefore required. DMA captures viscoelastic response and stiffness evolution, while NMR provides molecular-scale information on mobility and constraint distribution. When combined with transport measurements and microstructural observation, these techniques establish a framework linking diffusion behavior, viscoelastic response, and internal damage evolution.

This integrated framework is particularly important for hydrogen sealing applications, where degradation may occur through viscoelastic drift, internal cavitation, and loss of sealing force rather than immediate fracture. Reliable evaluation, therefore, requires combined transport, viscoelastic, molecular, and microstructural characterization under defined exposure conditions.

**Figure 6:**
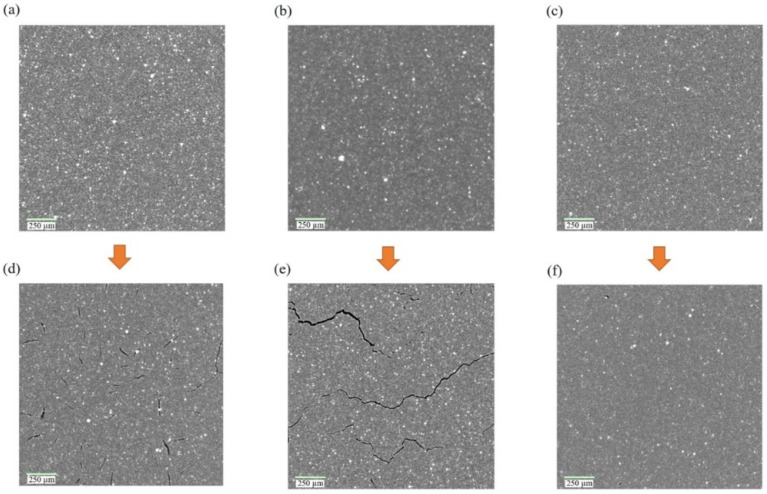
Micro–X-ray computed tomography (µXCT) images of EPDM elastomers illustrating formulation-dependent damage before and after high-pressure hydrogen exposure. (**a**–**c**) Unexposed samples: unfilled EPDM, plasticized EPDM, and carbon-black-filled EPDM. (**d**–**f**) Corresponding samples after hydrogen exposure. Unfilled and plasticized formulations exhibit internal cavitation and crack networks, whereas carbon-black-filled EPDM shows localized voids without continuous matrix cracking [7].

Figure 6 presents representative micro–X-ray computed tomography (µXCT) images of EPDM elastomers before and after hydrogen exposure [7]. The unexposed samples show relatively uniform internal structure, whereas exposed samples exhibit formulation-dependent damage. Unfilled and plasticized EPDM shows distributed cracking and larger cavities, indicating higher susceptibility to cavitation, while carbon-black-filled EPDM exhibits localized voids without continuous crack propagation, suggesting that filler reinforcement can restrict large-scale damage development.

Microstructural observations illustrate that hydrogen-induced changes vary significantly with elastomer formulation, with plasticized or weakly reinforced systems exhibiting greater susceptibility to swelling and cavitation, while reinforced materials tend to restrict bulk deformation but may still develop localized interfacial defects [7,20]. However, such observations alone do not fully resolve the underlying mechanisms governing material response.

This highlights the importance of polymer characterization in hydrogen systems. Microstructural features must be interpreted together with transport, viscoelastic, and molecular-scale measurements to establish a consistent understanding of material behavior across different length and time scales. Because distinct mechanisms—such as swelling, mobility redistribution, or internal damage—can produce similar macroscopic responses, reliance on a single technique may lead to ambiguous conclusions.

Accordingly, integrated characterization approaches are necessary to distinguish between reversible and irreversible changes and to relate observed structural features to transport and mechanical behavior. This is particularly critical for sealing applications, where performance degradation may arise from internal damage or viscoelastic evolution without visible surface failure, yet still result in loss of sealing integrity or leakage [12,20,22].

### 5.1. Experimental Protocol Considerations for High-Pressure Hydrogen Exposure

Evaluation of hydrogen-induced degradation in elastomers requires clearly defined experimental conditions, including pressure, temperature, exposure duration, specimen geometry, and decompression history [8,10,12]. Because these processes are strongly time- and temperature-dependent, experimental protocols must capture both transient and near-equilibrium responses. Aging studies typically cover pressures from approximately 10 to 90 MPa with exposure durations sufficient to approach saturation hydrogen uptake, together with extended times to evaluate swelling recovery, viscoelastic evolution, and compression-set development [10,12,19]. Both static aging and cyclic pressurization–depressurization procedures are relevant, as sealing materials in hydrogen infrastructure are subjected to repeated pressure variations during service [12,17]. Consistent comparison across studies requires standardized reporting of key experimental parameters, including exposure pressure, temperature, dwell time, decompression rate, specimen geometry, and post-exposure conditioning time. Variations in these parameters can significantly influence hydrogen uptake, diffusion behavior, and measured mechanical response. In particular, decompression rate plays a critical role in determining the severity of rapid gas decompression damage, while specimen thickness governs characteristic diffusion time scales. Standardized testing and reporting protocols are therefore essential for improving reproducibility and enabling reliable comparison of elastomer performance under hydrogen exposure conditions.

Building on these considerations, a comprehensive evaluation of elastomer performance requires systematic variation in both exposure and material parameters rather than single-condition testing. Experimental protocols should include a range of pressures (e.g., 10–100 MPa), controlled and rapid decompression conditions, and repeated pressurization–depressurization cycles to represent both service conditions and accelerated worst-case scenarios. Multi-pressure exposure sequences are particularly important, as transport and mechanical properties may not vary monotonically with pressure due to competing effects of swelling, constraint redistribution, and cavity formation.

In addition to exposure conditions, material-related variables—including crosslink density, filler type and loading, plasticizer content, and curing chemistry—must be considered, as these parameters govern diffusion behavior, swelling magnitude, viscoelastic response, and resistance to decompression damage. Hydrogen purity and potential contaminants may also influence sorption behavior and long-term material stability under service conditions. Temperature plays a critical role by simultaneously affecting hydrogen diffusivity and polymer relaxation times [8,10,15]. Accordingly, experimental matrices often include both ambient and elevated temperatures to accelerate transport processes, as well as lower temperatures near the glass-transition region where reduced chain mobility may increase hydrogen retention.

Decompression history must be explicitly defined, including pressure ramp rate, holding time, and venting procedure, because release conditions strongly influence cavitation, blister formation, and internal cracking [12,13]. Similarly, post-exposure conditioning is essential, as hydrogen desorption, dimensional recovery, and viscoelastic relaxation may continue after pressure release. The time interval between decompression and testing should therefore be reported to distinguish transient effects from more persistent material changes.

To isolate hydrogen-specific effects, control specimens aged under inert environments such as nitrogen or helium are often used [12,25]. However, inert gases do not fully reproduce hydrogen sorption and swelling behavior, and results obtained with surrogate gases should be interpreted cautiously. Because transport, swelling, and relaxation occur over different time scales, experimental protocols should include time-dependent evaluation of modulus evolution, damping behavior, and recovery after decompression. While conventional mechanical testing remains relevant for engineering qualification, it should be complemented by transport- and viscoelastic-based analysis to capture the multiscale nature of hydrogen-induced degradation.

Careful selection and reporting of pressure, temperature, exposure duration, decompression rate, and post-exposure conditioning are therefore essential for obtaining reproducible results and for establishing meaningful relationships between hydrogen transport, viscoelastic response, and long-term sealing performance in elastomers used for high-pressure hydrogen service [12,20,22].

### 5.2. Qualification and Standardization Considerations

Elastomer qualification procedures for hydrogen service are often adapted from conventional fluid-aging and decompression test methods developed for hydrocarbon or compressed-gas environments [12,20]. These approaches provide baseline evaluation of mechanical stability and decompression resistance but may not fully capture the time-dependent behavior governing performance in high-pressure hydrogen systems.

In hydrogen environments, gas uptake, swelling, and stress relaxation may occur on comparable time scales, and the resulting response depends strongly on exposure history, decompression rate, specimen geometry, and formulation. Consequently, evaluation based only on post-exposure tensile strength, hardness, or compression set may not fully represent in-service behavior. Similar mechanical properties alone are insufficient for hydrogen compatibility assessment [8,10,22].

A more comprehensive evaluation can be achieved through the integration of multiple characterization approaches. Transport measurements provide hydrogen uptake and diffusion time scales, DMA captures viscoelastic evolution, and controlled decompression testing evaluates resistance to cavitation. Imaging techniques such as micro–X-ray computed tomography (µXCT) or microscopy further reveal internal damage not visible at the surface [12,13,20].

Consistent reporting of exposure conditions is important for meaningful comparison. Pressure history, decompression rate, temperature, exposure duration, specimen geometry, and post-exposure conditioning time should be documented, as desorption, swelling recovery, and relaxation may continue after pressure release [8,10].

Several standards provide guidance for hydrogen service qualification, including ISO 19880-7 for hydrogen fueling systems and decompression-related testing, as well as CSA/ANSI CHMC 2 for hydrogen components and infrastructure [55,56]. These standards offer important frameworks for safety and performance evaluation; however, their scope is primarily focused on system-level considerations. As a result, additional attention to transport, viscoelastic, and microstructural aspects may be beneficial when assessing elastomer behavior under high-pressure hydrogen exposure.

Further development and harmonization of qualification approaches that incorporate transport, viscoelastic, and microstructural characterization could improve the reliability of hydrogen compatibility assessment, particularly for high-pressure storage, valve, and refueling systems where performance is influenced by diffusion–relaxation interactions during cyclic service [12,22].

### 5.3. Behavior of NBR and Fluoroelastomer Under High-Pressure Hydrogen Exposure

Nitrile butadiene rubber (NBR) and fluoroelastomers (FKM) are widely used sealing materials in high-pressure hydrogen systems and have been extensively studied under controlled exposure conditions. Experimental results show measurable swelling, increased compression set, residual strain, and reductions in rubbery storage modulus after exposure, particularly under cyclic pressures approaching 70–100 MPa [19,24]. These changes indicate time-dependent viscoelastic evolution rather than immediate fracture.

Hydrogen diffusivity in these materials is typically on the order of 10^−11^ m^2^/s, with EPDM generally showing higher diffusivity than NBR, and FKM exhibiting lower permeability but slower desorption [8,10]. Hydrogen uptake is commonly reported at ~400–600 wt ppm for NBR and EPDM and below ~200 wt ppm for FKM. However, swelling and mechanical response do not scale directly with hydrogen content, as they are strongly influenced by network structure and filler reinforcement [8,10].

Spectroscopic and NMR analyses indicate that the polymer backbone often remains largely intact after hydrogen exposure, with limited evidence of extensive chemical modification even under cyclic loading [20,24,25,45]. These observations suggest that hydrogen-induced changes are predominantly governed by physical processes such as swelling, increased chain mobility, and redistribution of network constraints.

NMR measurements further show hydrogen may exist in multiple mobility states within the elastomer network, including dissolved states in the polymer matrix and localized states within free-volume regions or filler–matrix interfaces. Such heterogeneous distribution can contribute to localized internal pressure development during decompression [41,45].

Microstructural observations using microscopy and µXCT reveal that internal voids, blistering, and crack formation may occur after decompression, with severity strongly dependent on formulation. Plasticized or weakly reinforced compounds generally exhibit greater swelling and more extensive damage, whereas filler-reinforced systems tend to limit crack propagation but may still develop localized defects [7,12,22].

DMA consistently shows reductions in storage modulus and increases in damping after hydrogen exposure in both NBR and FKM systems [15,19,24]. These changes are consistent with swelling-induced softening and modification of network constraints rather than extensive chemical degradation. Because such viscoelastic changes may occur without visible damage, gradual loss of sealing force can develop during cyclic service.

Overall, NBR and fluoroelastomers exhibit formulation-dependent changes in transport behavior, viscoelastic response, and microstructure during high-pressure hydrogen exposure while generally showing limited chemical modification. Reliable evaluation, therefore, requires combined analysis of transport, viscoelastic, molecular, and microstructural data to interpret long-term sealing performance [10,12,22].

### 5.4. Engineering Framework for Hydrogen Compatibility Assessment

To translate the discussed mechanisms into practical engineering applications, a structured evaluation framework for hydrogen compatibility is proposed. First, hydrogen transport properties, including diffusivity and solubility, should be quantified to determine the rate of gas ingress and total uptake [8,12]. Second, the characteristic diffusion time should be evaluated relative to component thickness to assess whether equilibrium conditions can be achieved during service [12,13]. Third, viscoelastic properties should be characterized using techniques such as DMA to understand how hydrogen exposure affects polymer mobility and relaxation behavior [20,25,30].

In addition, the role of fillers and crosslink density must be evaluated, as these factors directly influence both diffusion pathways and mechanical response [8,10]. Susceptibility to rapid gas decompression (RGD) can then be assessed by considering the relative balance between hydrogen transport and viscoelastic relaxation [12,13]. Materials that exhibit slower pressure equilibration relative to their ability to relax stress are more prone to transient internal pressure buildup and cavitation, whereas systems that allow faster equilibration tend to show more stable behavior.

From a practical standpoint, elastomers combining high hydrogen solubility with low diffusivity are more susceptible to gas retention and stress accumulation, while moderate diffusivity with lower gas uptake promotes pressure equalization. Furthermore, increased filler content and crosslink density enhance dimensional stability but may also introduce heterogeneous transport pathways and localized stress concentration [8,10]. These considerations provide a practical basis for linking transport behavior, viscoelastic response, and formulation characteristics to material selection and seal design in hydrogen applications [12,20,22].

## 6. Qualification Considerations and Research Directions

Qualification procedures for elastomeric seals in hydrogen service are commonly adapted from conventional fluid-aging and decompression test methods developed for hydrocarbon or compressed-gas applications [12,20]. While these approaches provide baseline evaluation of mechanical stability and decompression resistance, they do not fully capture swelling and viscoelastic effects governing elastomer behavior in high-pressure hydrogen environments [8,10,22]. In such conditions, gas uptake, stress relaxation, and mechanical evolution may occur on comparable time scales, with response strongly influenced by exposure history, decompression rate, and formulation-dependent transport properties.

Across published studies, qualification protocols vary in the extent to which hydrogen transport, viscoelastic response, and decompression conditions are quantified [8,10,22]. Evaluation based solely on post-exposure tensile strength or hardness may not represent material performance, as conventional properties may not reflect hydrogen response.

Formulation variables, including crosslink density, filler reinforcement, cure chemistry, and plasticizer content, play a dominant role in hydrogen response [12,13,24]. Decompression history and cyclic pressure loading further influence damage evolution through diffusion–relaxation effects, as discussed in Section 3.3 [12,13,32].

Meaningful comparison between studies, therefore, requires clear reporting of specimen geometry, exposure pressure, temperature, dwell time, decompression protocol, and post-exposure conditioning [8,10]. Hydrogen desorption, dimensional recovery, and viscoelastic relaxation may continue after pressure release, and differences in conditioning time can significantly affect measured properties. In addition, transport behavior in heterogeneous elastomers may deviate from ideal diffusion, limiting the direct transferability of parameters across different conditions.

Future work would benefit from systematic coupling of hydrogen transport measurements with mechanical, spectroscopic, and microstructural characterization. In addition, data-driven and predictive modeling approaches, including machine learning techniques, have shown potential for correlating experimental parameters with sealing performance and may support the development of more reliable qualification methodologies [57].

Development of standardized methodologies incorporating transport parameters, relaxation behavior, and controlled decompression protocols will be important for hydrogen storage, refueling, and valve-sealing applications. Such approaches may enable more consistent evaluation of elastomer performance by accounting for the interaction between diffusion, swelling, viscoelastic response, and microstructural stability under high-pressure hydrogen exposure [10,12,22].

## 7. Conclusions

Hydrogen environments impose demanding service conditions on elastomeric sealing materials due to elevated pressure, repeated pressurization–depressurization cycles, and the strong coupling between gas transport and viscoelastic relaxation of polymer networks. Unlike conventional fluid exposure, hydrogen penetrates elastomers primarily through solution–diffusion processes and may accumulate in free-volume regions, filler–matrix interfaces, or locally constrained domains within the polymer network. This sorption-driven process produces swelling, redistribution of molecular mobility, and development of internal stresses that depend on both transport kinetics and viscoelastic relaxation behavior. Under rapid decompression, transient internal pressure gradients may develop, as discussed in Section 3.3. As a result, degradation in hydrogen environments is governed primarily by diffusion–relaxation interactions and formulation-dependent network response rather than by fracture-controlled mechanisms typical of metallic materials.

Experimental studies on commonly used sealing elastomers such as NBR, HNBR, FKM, EPDM, and silicone indicate that limited chemical modification is generally observed after high-pressure hydrogen exposure, while measurable changes occur in swelling, modulus, damping behavior, and internal morphology. These observations suggest that hydrogen-induced degradation is predominantly governed by physical processes, including gas sorption, free-volume expansion, redistribution of viscoelastic constraints, modification of filler–matrix interactions, and decompression-driven cavity growth, although localized chemical modification may occur under severe exposure conditions. Because filled and heterogeneous elastomers may exhibit non-ideal or spatially non-uniform diffusion behavior, hydrogen can exist simultaneously in multiple mobility states, and localized gas retention may produce non-uniform internal pressure during decompression. Resistance to damage therefore depends not only on total hydrogen uptake but also on its spatial distribution and on the ability of the polymer network to relax internal stresses during pressure changes.

A consistent finding across the literature is that hydrogen compatibility cannot be evaluated using a single property. Techniques such as dynamic mechanical analysis, NMR characterization, permeation measurements, controlled decompression testing, and microstructural imaging provide complementary information on molecular mobility, network constraints, gas transport, and macroscopic mechanical stability. The combined use of these methods enables correlation of diffusion kinetics, viscoelastic relaxation, and structural evolution, allowing more reliable interpretation of formulation-dependent degradation behavior. Such multiscale characterization is particularly important because similar changes in stiffness or swelling may result from reversible plasticization, filler-network modification, or irreversible microstructural damage.

Material formulation plays a dominant role in hydrogen response. Crosslink density, filler reinforcement, plasticizer content, cure chemistry, and specimen geometry influence hydrogen solubility, diffusivity, swelling magnitude, viscoelastic stability, and resistance to decompression damage. Exposure pressure, temperature, decompression rate, and cycling history further control the competition between diffusion and relaxation time scales, and experimental results may exhibit non-monotonic dependence on pressure due to competing effects of swelling, constraint redistribution, and cavity nucleation. Reliable qualification of elastomer seals for hydrogen applications therefore requires systematic evaluation across a wide range of pressures, temperatures, exposure durations, and material formulations rather than testing at a single condition.

From a seal design perspective, the performance of elastomers in hydrogen environments is governed by the balance between transport behavior and mechanical stability. Materials with low diffusivity may retain hydrogen and increase the risk of internal pressure buildup during decompression, while high solubility leads to greater gas uptake and potential damage. Filler reinforcement improves stiffness and dimensional stability but introduces heterogeneity that may act as both diffusion barriers and trapping sites. Therefore, optimal material selection requires balancing transport properties, viscoelastic response, and formulation characteristics to minimize hydrogen-induced degradation while maintaining sealing performance.

Future studies should emphasize well-defined experimental protocols that include multi-pressure exposure, controlled decompression procedures, elevated-temperature testing, and comparison of immediate and delayed measurements after pressure release. Such approaches are necessary to distinguish reversible transport-induced effects from irreversible network damage and to reduce inconsistencies between reported results. Improved reporting of material composition, exposure history, and testing conditions will be essential for meaningful comparison between studies, particularly for filled elastomers where heterogeneous diffusion and filler–polymer interactions strongly influence behavior.

Development of reliable qualification methods for hydrogen sealing materials will require testing frameworks that integrate transport measurements, viscoelastic characterization, spectroscopic analysis, and microstructural observation under controlled conditions. Establishing relationships between transport behavior, filler-network stability, and decompression response will be critical for defining predictive criteria for long-term sealing performance. Such multiscale and formulation-sensitive approaches are expected to play an important role in the safe design of hydrogen storage, refueling, and high-pressure energy systems, where sealing reliability depends on the interaction between gas transport, viscoelastic evolution, and microstructural stability rather than on chemical resistance alone. From a materials selection perspective, elastomers with low hydrogen solubility, moderate-to-high diffusivity, and stable filler-reinforced networks are expected to provide improved resistance to decompression damage and long-term sealing degradation.

## Figures and Tables

**Table 1:** Representative environmental parameters reported in hydrogen exposure studies of elastomers and their relevance to degradation behavior.

Parameter	Typical Range Reported in Hydrogen Studies	Influence on Hydrogen Transport and Degradation Mechanisms	Refs
Pressure	~1–90 MPa in laboratory studies; up to ~100 MPa in storage/refueling systems	Controls hydrogen solubility (S) and uptake; higher pressure increases absorbed gas, swelling, and internal pressure during decompression, increasing RGD risk	[7,12,13]
Temperature	−40 to 80 °C service; up to 100–120 °C accelerated aging	Affects diffusivity (D), solubility (S), and viscoelastic relaxation; higher temperature increases transport rate and swelling, lower temperature increases stiffness and internal stress	[18,19,20]
Exposure duration/dwell time	Seconds to hundreds of hours; cyclic tests up to 10^3^–10^5^ cycles	Determines approach to sorption equilibrium and time-dependent relaxation; long exposure increases uptake, repeated cycles promote microstructural evolution and stiffness drift	[3,15]
Pressure cycling history	Single exposure to repeated pressurization–decompression cycles	Produces cyclic swelling and diffusion gradients; may cause network relaxation, filler–matrix debonding, and progressive mechanical changes without visible cracks	[5,9]
Decompression rate	Seconds (rapid venting) to minutes/hours (controlled release)	Governs internal pressure gradient; rapid decompression may cause supersaturation, cavitation, blistering, and crack growth; slow release reduces RGD damage	[5,9,21]
Gas composition	Pure H_2_; He, N_2_, CH_4_ or mixtures for comparison	Gas type affects solubility, diffusivity, and permeability; hydrogen shows high diffusivity and strong mobility effects not always reproduced by surrogate gases	[20,22]
Polymer formulation variables	Crosslink density, ACN content, filler loading, plasticizer, cure system	Controls S, D, modulus, and cavitation resistance; high crosslink density reduces swelling, fillers may lower permeability but create local stress concentrations	[9,18,23]
Specimen geometry/thickness	~1–3 mm membranes; bulk samples/O-rings up to >10 mm	Diffusion time ∝ L^2^/D; thicker samples retain hydrogen longer, increasing internal pressure during decompression	[3,20]
Post-exposure conditioning	Immediate testing to hours/days after exposure	Desorption continues after decompression; measured properties depend on remaining hydrogen and viscoelastic recovery	[3,15]
Permeability/diffusivity/solubility	P ≈ 10^−10^–10^−8^ mol·m/(m^2^·s·Pa); D ≈ 10^−11^–10^−9^ m^2^/s	Transport parameters control uptake and release; high S and low D increase trapped gas and RGD risk, higher D promotes faster gas escape	[19,20,22]
Viscoelastic properties	Modulus, Tg, loss factor depend on formulation and temperature	Hydrogen uptake modifies chain mobility and free volume, affecting stress relaxation, stiffness, and sealing force retention	[8,15,18]

**Table 2:** Experimentally reported degradation mechanisms in elastomers exposed to high-pressure hydrogen and their relevance to seal performance.

Degradation Mechanism	Polymer-Level Response	Seal-Level Consequence	Key Methods	Elastomers	Refs
Sorption-induced swelling	Free-volume expansion, reversible softening	Reduced contact stress, sealing-force loss	Sorption, swelling, DMA	NBR, EPDM, FKM, silicone	[19,20,22]
Rapid gas decompression (RGD)	Internal pressure gradients → cavitation, void nucleation	Blistering, cracks, leakage, failure	µXCT, microscopy	EPDM, NBR, filled elastomers	[5,9,21]
Delayed desorption	Time-dependent gas release, modulus recovery	Temporary sealing-force loss	TDA, swelling recovery, DMA	NBR, HNBR, FKM	[3,15]
Viscoelastic evolution	Increased mobility, reduced modulus, higher damping	Stress relaxation, compression set, leakage	DMA, stress relaxation	NBR, EPDM, FKM, silicone	[8,15,18]
Elevated temperature effects	Accelerated diffusion and relaxation, enhanced softening	Faster stiffness loss	DMA, DSC/TGA	NBR, HNBR, FKM	[18,19]
Formulation-dependent effects	Filler debonding, microvoid formation, reduced reinforcement	Higher compression set, leakage risk	µXCT, SEM, DMA	Filled NBR, EPDM, HNBR	[9,18,23]
Low-temperature near Tg	Reduced mobility, stress accumulation	Brittle response, crack initiation	DMA low-T, fracture tests	High-Tg elastomers	[20,21]
Diffusion–relaxation mismatch	Gas release slower than pressure drop → stress buildup	Cavitation, cracks, stiffness drift	Decompression, µXCT, DMA	NBR, EPDM, FKM	[5,8,9]

**Table 3:** Hydrogen sorption and diffusion properties of common elastomeric seal materials. Adapted from multiple sources.

Polymer	Pressure (MPa)	Temp (°C)	Equilibrium H_2_ Content (wt ppm)	Diffusivity D (×10^−11^ m^2^/s)	Relative Permeability Trend	References
NBR	~90	25	450–500	2–3	Moderate–High	[19,24,26]
EPDM	~90	25	~550	3–4	High	[9,19,24]
FKM	~90	25	90–150	1–2	Low	[18,24]
HNBR	~90	25	~150	~1	Low	[19,24]

**Table 4:** Methods commonly used for interpretation of viscoelastic and mechanical changes in hydrogen-exposed elastomers. These approaches help distinguish reversible plasticization, diffusion-controlled stress development, and irreversible microstructural damage during high-pressure hydrogen exposure [8,10,11,15,24,34].

Method	Purpose	Physical Meaning in Hydrogen Exposure
Relaxation spectrum/Maxwell analysis	Determines relaxation-time distribution	Indicates redistribution of relaxation processes due to hydrogen sorption and free-volume changes
Time–temperature superposition (TTS)	Relates frequency to temperature-dependent mobility	Identifies Tg shift and mobility changes associated with plasticization
Storage modulus (E′)	Measures stiffness	Reduction indicates softening due to swelling or weakened filler interactions
Loss modulus/tan δ	Evaluates damping	Increase reflects higher mobility or filler-network disruption
Swelling/volume change	Quantifies hydrogen uptake	Retained hydrogen may generate internal pressure during decompression
Diffusion–relaxation comparison (τ diff vs. τ relax)	Compares transport and relaxation rates	Mismatch leads to pressure gradients and cavitation risk
NMR analysis	Probes molecular mobility and constraints	Distinguishes reversible plasticization from structural modification
Microstructural observation (SEM/µCT)	Detects internal defects	Confirms cavitation, microvoids, or interfacial damage
Filler-network evaluation	Assesses reinforcement integrity	Explains stiffness drift and damping increase
Cyclic pressurization testing	Evaluates durability	Reveals progressive viscoelastic evolution and functional degradation

**Table 5:** Experimental and reporting considerations for reliable dynamic mechanical analysis of hydrogen-exposed elastomers [15,25,34,35,36,37].

Category	Recommended Practice/Parameter	Relevance to Hydrogen Exposure
Deformation mode	Consistent geometry and loading.	Swelling alters stress distribution.
Strain amplitude/LVE	Verify linear viscoelastic region.	Hydrogen may increase strain sensitivity.
Frequency range	Report frequency	Relaxation behavior may shift after sorption.
Temperature control	Maintain constant temperature.	Diffusion and relaxation are temperature-dependent.
Pressure history	Report pressure, cycles, decompression.	Controls transport–relaxation balance.
Sample state	Time after decompression, storage.	Desorption affects measured response.
Specimen geometry	Thickness and shape.	Diffusion time ∝ L^2^/D.
Material formulation	Polymer, filler, crosslink density.	Governs transport and viscoelastic behavior.
Data interpretation	Tg method and analysis approach.	Distinguishes plasticization vs. structural change.
Replication	Report variability.	Diffusion and damage may be non-uniform.
DMA response	E′, E″, tan δ vs. T/frequency.	Quantifies viscoelastic stability.

**Table 6:** Experimental techniques used to evaluate hydrogen-induced degradation in elastomers and typical observations reported in high-pressure hydrogen studies [36,37,41,43,44].

Method	Property Measured	Typical Observation	Interpretation
DMA	Storage modulus, damping	Reduced modulus, increased damping	Mobility redistribution, swelling, filler effects
NMR	Molecular mobility, hydrogen uptake	Changes in relaxation behavior	Hydrogen in free volume and interfacial regions
Decompression/RGD	Cavitation, cracking	Voids, cracks after pressure release	Diffusion–relaxation mismatch
µXCT/microscopy	Internal morphology	Cavities, microcracks	Formulation-dependent damage
Swelling/desorption	Hydrogen uptake, volume change	Swelling and delayed recovery	Transport-controlled sorption/desorption

## Data Availability

No new data were created or analyzed in this study.
